# Evaluating radiofrequency electromagnetic field exposure in confined spaces: a systematic review of recent studies and future directions

**DOI:** 10.1093/rpd/ncae045

**Published:** 2024-03-15

**Authors:** Muhammad Ahsan Ashraf, Turgay Celik

**Affiliations:** Sibanye-Stillwater Digital Mining Laboratory (DigiMine), University of the Witwatersrand, Johannesburg 2000, South Africa; School of Electrical and Information Engineering, Faculty of Engineering and Built Environment, University of the Witwatersrand, Johannesburg 2000, South Africa; School of Electrical and Information Engineering, Faculty of Engineering and Built Environment, University of the Witwatersrand, Johannesburg 2000, South Africa; Faculty of Engineering and Science, University of Agder, 4630 Kristiansand, Norway

## Abstract

This study reviews recent research on Radiofrequency Electromagnetic Field (RF-EMF) exposure in confined environments, focusing on methodologies and parameters. Studies typically evaluate RF-EMF exposure using an electric field and specific absorption rate but fail to consider temperature rise in the tissues in confined environments. The study highlights the investigation of RF-EMF exposure in subterranean environments such as subways, tunnels and mines. Future research should evaluate the exposure of communication devices in such environments, considering the surrounding environment. Such studies will aid in understanding the risks and developing effective mitigation strategies to protect workers and the general public.

## Introduction

Modern wireless communication technologies have become ubiquitous in modern society^(1)^, with applications ranging from personal devices^(2)^ and machine-to-machine communication^(3)^ to connected vehicles^(4)^, Internet of Things systems^(5)^ and healthcare solutions for Industry 4.0^(6)^. These technologies operate on electromagnetic fields (EMFs) within the radiofrequency (RF) range. In confined spaces, such as subways, tunnels and mines, the proximity of wireless devices and the increased reflection of EMFs can lead to higher exposure levels for humans. As a result, concerns have been raised about potential health effects from Radiofrequency Electromagnetic Field (RF-EMF) exposure in confined environments. Various standard bodies, such as the International Commission on Non-Ionizing Radiation Protection (ICNIRP)^(7)^ and the Institute of Electrical and Electronics Engineers (IEEE)^(8, 9)^, have established exposure limits for RF-EMF in terms of electric field (volt (V)/meter (m)), power density (PD, Watt meter$^{-2}$), specific absorption rate (SAR, Watt (W)/kilogram (kg)) and temperature rise in tissues. The International Agency for Research on Cancer has also classified RF-EMF as a Group 2B ‘possible carcinogen’ to humans^(10)^. Previous research has reported various health effects associated with RF-EMF exposure^(11, 12, 13, 14)^, including physiological effects such as headaches, brain tumors and dizziness when SAR limits are exceeded^(11)^. Given the potential risks, thoroughly investigating RF-EMF exposure in confined and reflective environments is crucial.

Several literature reviews have summarized the studies of RF-EMF exposure in contexts of different confined and occupational environments. Sagar *et al.* systematically reviewed the spatial distribution of RF-EMF exposure in the everyday environment in Europe by summarizing 21 studies conducted in European countries between 2000 and 2013^(15)^. Chiaramello *et al.* reviewed research on RF-EMF exposure in indoor environments and found that mean values of whole RF-EMF frequency bands were higher in offices and public transports, while low levels of exposure were observed in homes and apartments^(1)^. Different assessment strategies have been used to measure RF-EMF exposure in confined environments, such as personal exposure meters (PEMs) and smartphone-based applications^(16)^, but a standard procedure has yet to be established. Moreover, with the implementation of 5G technology, it is critical to update literature reviews to include recent research and assessment strategies in confined environments, including 5G technologies.

Gallucci *et al.* reviewed studies related to RF-EMF exposure in occupational military scenarios. They found that exposure varied depending on the source of RF-EMF exposure, and further studies are necessary to assess exposure for specific devices and technologies such as 5G devices, especially in military scenarios^(17)^. In recent years, there has been a surge of interest in computational dosimetric studies on RF-EMF exposure above 6 GHz. Hirata *et al.* reviewed several studies on steady-state and transient temperature rise due to sinusoidal exposure, highlighting the use of absorbed/epithelial PD as a new dosimetric parameter, which has been adopted in international guidelines/standards for RF-EMF exposure to humans above 6 GHz^(18)^.

Kim *et al.* reviewed studies on RF-EMF exposure from various commercial wearable communication devices and pointed out that wearables can cause higher levels of SAR at the skin surface due to their direct physical contact or extreme proximity to the human skin^(19)^. The general public needs to be informed about the exposure to the latest commercial wearable devices, updating safety regulations and educating manufacturers about the latest research and regulations. Abdul-Al *et al.* provided valuable insights into RF-EMF interaction with biological media, highlighting the importance of SAR in understanding the exposure with induced field intensity, human tissues and their dielectric properties^(20)^. Similarly, Orzeszyna *et al.* also estimated PD and SAR for RF-EMF exposure in trains, elevators and cars by applying multimode resonant cavity theory^(21)^. Simultaneously, using several mobile phones exceeds the ICNIRP guidelines in such confined environments.

Benke *et al.* reviewed the long-term RF-EMF exposure on cognition in humans systematically, highlighting the need for continued research in understanding the effects of RF-EMF exposure on human health^(22)^. Guido *et al.* reviewed the design of antennas for wearable and implantable applications, aiming to reduce the SAR while keeping the same radiation characteristics^(23)^. Similarly, different SAR reduction techniques were reviewed, which identified several techniques such as Photonic Band Gap, Electromagnetic Band Gap and split ring resonator for reducing SAR^(24)^. Overall, these reviews emphasize the need for continued research to understand the effects of RF-EMF exposure on human health and identify effective measures to reduce SAR. It is also essential to inform the general public, update safety regulations and educate manufacturers about the latest research and regulations.

The assessment of RF-EMF exposure has become increasingly important due to the growing number of electronic devices and wireless communication technologies. However, assessing RF-EMF in confined environments such as personal vehicles, planes, subways, buses, elevators and industrial environments poses unique challenges due to limited space and nearby metallic objects. Therefore, a systematic literature review (SLR) is necessary to summarize the current state of knowledge. This study aims to provide an up-to-date and comprehensive review of recent research on RF-EMF exposure in confined environments for the last 5 years. The systematic review process is outlined in Section II, while the selected studies are analyzed in Section III. Current challenges and emerging directions for RF-EMF exposure in confined environments are discussed in Section V. Finally, Section VI concludes the study.

## Systematic review process

The following steps were followed for the systematic literature survey^(25)^.

### Development of protocol

A predefined protocol was developed to eliminate the feasibility of bias, consisting of the following steps:

1. Framing of research questions.
2. Strategy for searching databases.
3. Identification of Inclusion and Exclusion criteria.
4. Assessing quality assessment (QA) of the articles.
5. Information extraction from the articles.

### Research questions

The following questions were identified to analyse the RF exposure in the confined environment of the underground mine:

1. What methods and techniques evaluate RF-EMF in confined environments?
2. What parameters are used to evaluate RF-EMF in confined environments?
3. Which factors are involved in RF-EMF exposure value in confined environments?
4. How temperature elevation in the tissues is evaluated in confined environments?
5. What factors should be included to analyse the SAR and temperature elevation in the tissues for the sub-terrain environment?

### Search strategy

The primary papers were systematically identified after extensive research, and articles related to research questions were selected. The details of search strategies are provided below.

#### Database search

For this review, articles on RF-EMF exposure in confined environments were searched in digital databases for grey literature, including conference proceedings, unpublished articles and books. The following most relevant digital databases were searched:

1. Google Scholar
2. Research Gate
3. Institute of Physics
4. ScienceDirect
5. IEEE Xplore
6. Multidisciplinary Digital Publishing Institute

#### Search terms

A broad set of search terms, such as ‘specific absorption rate’ OR ‘electromagnetic exposure’ OR ‘mobile phone exposure’ OR ‘radiofrequency exposure’ OR ‘exposure assessment’ AND ‘enclosed environment’ OR ‘metallic object’ OR ‘vehicle’ OR ‘indoor environment’ OR ‘elevator’ AND ‘exposimeter’ AND ‘temperature elevation’, and appropriate papers were selected.

### Screening process

The primary goal of the screening process was to choose applicable papers from the total searched database to answer the research questions. In addition, the likelihood of the bias was removed by introducing the inclusion and exclusion criteria.

#### Inclusion criteria

Initial screening was carried out based on the title and abstract, but the final inclusion was done after reading the full text of the articles. As a result, the articles passed the following criteria and were included for further study:

- Articles published between 1 January 2018 and 10 March 2023.
- Articles investigate RF-EMF exposure in vehicles, subways, busses, planes, elevators and indoor environments.
- Articles investigate the impact of metal objects near human tissues along with a source of RF-EMF.
- Articles published in English language and fulfill the criteria.

#### Exclusion criteria

The following criteria were set to exclude the non-eligible articles:

- Articles investigated RF-EMF exposure in the outdoor environment and focused on other parametric studies.
- Articles investigate the SAR and temperature elevation due to implantable devices and antennas without considering any surrounding environment.
- Articles investigate exposure at extremely low frequencies, such as exposure to electrical appliances and charging of electric vehicles.
- Articles provide general conclusions without added value to the topic.
- Articles included in the category: newsletter, editorial, a summary of the workshop, technical interview session summary and preface.
- Articles with the unavailability of the full text.

### Quality assessment

Quality assessment (QA) is an appropriate tool for selected articles to conduct a more detailed study. It is employed to rate the selected articles and significantly reduces the bias in selecting the articles^(26)^. The QA was completed by setting up questions, and each primary selected article was assessed through those questions. All the questions were evaluated on a scale of 0–2 (0 means unsatisfied, 1 means partially satisfied and 2 means satisfied). Table 1 shows the set of formulated QA questions. The articles whose total QA scores were above and equal to 7 against all the questions were considered primary papers for this SLR.

**Table 1:** Formulation of QA questions for SLR

	Question
QA1	To what extent does the article cover the research question?
QA2	Does the paper body meet the problem covered in the abstract?
QA3	How well methodology used in the study is explained?
QA4	Does the article compare the results with other studies or methodologies?
QA5	Did the article mention the limitations of the study?

### Data extraction

Systematically, the information was collected from primary papers. Data were collected, which addressed the issues of the review and QA. The following data were collected from primary papers in the data extraction:

1. Date of data extraction;
2. Title of the article;
3. Name of author(s);
4. Problem(s) addressed;
5. Concluding remarks and
6. QA score.

### Review result

**Figure 1:**
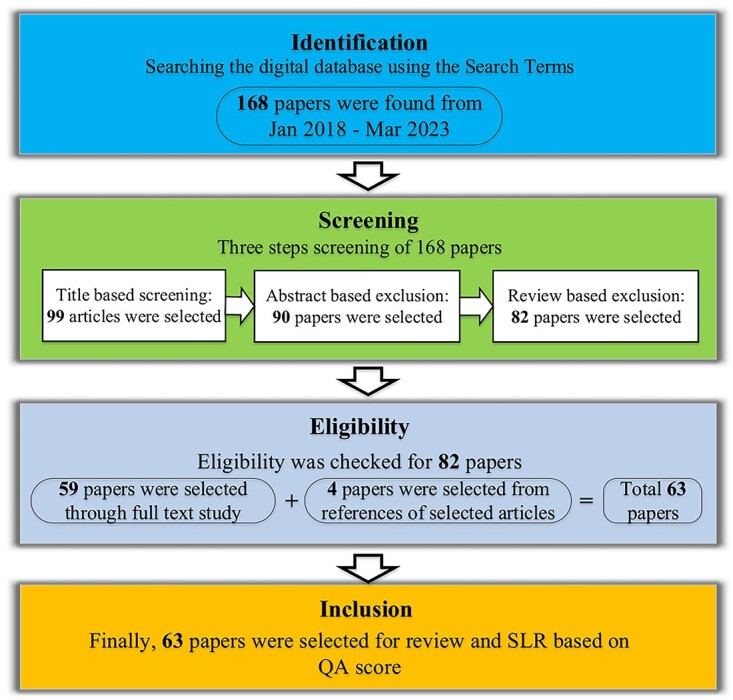
The SLR process.

Identifying potential articles through a systematic search was compiled by selecting the primary articles. The selection process of primary papers is shown in Figure 1. The screening and eligibility criteria were applied to the identified articles, and qualified articles were considered in the next steps. In the last step, articles that met eligibility criteria were appraised to QA.

## Detailed Review and analysis

### Selection of studies

The literature survey identified 168 potentially relevant articles using the specified search terms. After applying inclusion criteria, 105 articles were excluded, as illustrated in Figure 1. The remaining articles were categorized into two groups based on their methodologies: measurement and simulation. Only five articles employed measurement and simulation methods to evaluate RF-EMF exposure in various confined environments. A detailed analysis of the selected articles is presented below.

### Dosimetry measurements

A total of 39 selected articles utilized different dosimetric measurement methods to assess RF-EMF exposure. These articles are further divided into indoor (dwelling) and confined environments, including subway trains, buses, cars, aircraft, tunnels, elevators and mines. Table 2 summarizes the studies that assessed RF-EMF exposure through measurement in different indoor and confined environments for various frequencies and scenarios. In this study, selected studies are denoted by small parentheses ’()’ as listed in Tables 2 and 2, and citation sources are indicated by square brackets ’[]’.

**Table 2:** List of selected studies performed dosimetric measurements

ID	Study	Equipment	Parameter	Microenvironment	Country	QA	Source
1	Choi *et al.*	ExpoM-RF	Power density	Home, school, office, car, metro and sports centres	South Korea	8	^(27)^
2	Karpowicz *et al.*	EME SPY 200	Electric field	Offices, schools, libraries, public transport, and shopping centers	Spain	8	^(28)^
3	Zeleke 2018 *et al.*	ExpoM-RF	Electric field	Residential, school and office	Australia	7	^(29)^
4	Hardell *et al.*	EME SPY 200	Power density	Rooms of the apartment	Sweden	7	^(30)^
5	Zeleke *et al.* 2019	Questionnaire and ExpoM-RF	Electric field and Linear regression	Residence near mobile base station in the metropolitan areas of Melbourne	Australia	8	^(31)^
6	Massardier-Pilonchery *et al.*	EME SPY 200	Electric field	Libraries and Media Libraries	France	7	^(32)^
7	Ramirez-Vazquez *et al.* 2019a	EME SPY 140	Power density	Different 14 microenvironments such as buildings, shopping malls, hospital, schools, universities, home, office and in the public transport	Spain	9	^(33)^
8	Ramirez-Vazquez *et al.* 2019b	EME SPY 140	Power density	Albacete Fair (large public event)	Spain	9	^(34)^
9	Iyare *et al.* 2019	Dipole antenna and spectrum analyzer (Anritsu MS2721A)	Electric field	City center of Leuven	Belgium	8	^(35)^
10	Kiouvrekis *et al.*	Spectrum analyzer SRM 3006 with tri-axial antenna	Electric field	Greek primary and secondary education schools located in urban environments	Greece	8	^(36)^
11	Loh *et al.*	Tri-axial isotropic field probe and spectrum analyser (FieldFox N9917B)	Electric field	Basement of the 5G Innovation Centre (5GIC) at the University of Surrey	England	7	^(37)^
12	Paniagua *et al.*	EME SPY 200	Electric field	Inside and outside dwelling (buildings, flat and apartment) in the city of Cáceres	Spain	8	^(38)^
13	Keshmiri *et al.*	SMP (Exposimeter) and probes	Electric field and power density	Corridors, indoor and outdoor locations of Ferdowsi University of Mashhad	Iran	7	^(39)^
14	Moraitis *et al.*	NARDA SRM- 3000	Electric field and power density	24 different corporate building of the Athens city center	Greece	7	^(40)^
15	Mannan *et al.*	HF-B3G (RF meter)	Electric field, magnetic field and power density	Residential, hospital, office, and school and entertainment centers inside Qatar Foundation	Qatar	8	^(41)^
16	Ramirez-Vazquez *et al.* 2020	EME SPY 140	Power density	Inside and outside school	Spain	7	^(42)^
17	Onishi *et al.*	Spectrum analyzer and SRM-3006 NARDA	Electric field	Outdoor and underground shopping malls	Japan	8	^(43)^
18	Iyare *et al.* 2021	Near field probe and spectrum analyzer (Keysight N9344C)	Electric field	Indoor environment of different city centres of the Leuven	Belgium	8	^(44)^
19	Ramirez-Vazquez *et al.* 2021	EME SPY 140	Power density	Exposure of 63 volunteers from home, workplace, outside, schools, travel, and shopping	Mexico	9	^(45)^
20	Kapetanakis *et al.*	NARDA SRM-3006	Electric field	Inside schools	Greece	7	^(46)^
21	Rathebe *et al.*	TM-196 3-Axis RF-field strength meter	Power density	Residential buildings	South Africa	7	^(47)^
22	Bakcan *et al.*	NARDA SRM 3006	Electric field	University campus	Turkey	7	^(48)^
23	Schmutz *et al.*	ExpoM-RF	Power density	School and Home	United Kingdom	7	^(49)^
24	Hamiti *et al.*	EME SPY-140	Power density	School	Kosovo	8	^(50)^
25	Ramirez-Vazquez *et al.* 2023	EME SPY 140	Power density	Campus area of the University	Spain	7	^(51)^
26	Michalowska *et al.*	ESM-140	Electric field and SAR	CNC machine area	Poland	7	^(52)^
27	Cerezci *et al.*	Narda ELT-400, HOLADAY 3060, Narda 550, Narda SRM-3006	Electric and magnetic field	Cleaning product manufacturing factory of 400 workers	Turkey	8	^(53)^
28	Wafik *et al.*	Electrosmog meter Cornet	Volt/meter and $\mu $Tesla	Inside Office of the building, New York	USA	7	^(54)^
29	Sagar *et al.*	ExpoM-RF	Electric field and Power density	94 indoor and 18 outdoor areas (public transport)	Switzerland, Ethiopia, Nepal, South Africa, Australia, and USA	8	^(55)^
30	Gryz *et al.*	EME SPY 121 and NARDA SRM 3006	Electric field	Trains and TESTSAFE-BIO laboratories	Poland	8	^(56)^
31	Gombarska *et al.*	Spectrum analyzer (BK2650A)	Electric field	Inside personal vehicle	-	8	^(57)^
32	Sen *et al.*	dipole antenna and spectrum analyzer	Electric field, penetration depth and absorbed power	An-echoic chamber with radiation absorbent material	-	8	^(58)^
33	Schilling *et al.*	NARDA SRM 3006	Electric field reference level of ICNIRP %	Vehicle	-	8	^(60)^
34	Vaverka *et al.*	Spectrum analyzer and dipole antenna	Electric field	Shielded area	-	8	^(61)^
35	Aminzadeh *et al.*	Body worn distributed meter, ExpoM-RF and spectrum analyzer	Power density	Corridors and offices	-	9	^(62)^
36	Huss *et al.*	A multi-band body-worn distributed-exposimeter integrated in the vest, ExpoM-RF and EME SPY 200	Power density	Shopping areas, train stations, outdoor rural/ urban residential environments	Belgium, Spain, France, the Netherlands and Switzerland	9	^(63)^
37	Bustillo *et al.*	NARDA SRM-3000	Average power density	Philippine General Hospital (PGH)	Philippine	7	^(64)^
38	Martínez-Gonzalez *et al.*	NARDA NBM-550 broadband field meter	Electric field	Library reading room	Spain	7	^(65)^
39	Kljajic *et al.*	NARDA NBM-550	Electric field and global exposure ratio (GER)	Campus area of the University of Novi Sad	Serbia	7	^(66)^
40	Bejenaru *et al.* 2019	CST Microwave Studio	SAR	Inside building	FIT	8	^(68)^
41	Psenakova *et al.*	CST Microwave Studio	SAR	Inside shielded space	FIT	7	^(69)^
42	Ashraf *et al.*	CST Microwave Studio	SAR	Underground mine	FIT	7	^(70)^
43	Chiaramello *et al.*	WiCa Heuristic Indoor Propagation Prediction (WHIPP)	Electric field	Residential	Kriging method	8	^(71)^
44	Shikhantsov *et al.*	REMCOM	SAR	Industrial indoor environment	Ray-tracing method and FDTD	8	^(72)^
45	Bejenaru *et al.* 2020	CST Microwave Studio	SAR	Indoor room with different materials	FIT	7	^(73)^
46	Bhargava *et al.*	COMSOL Multiphysics	SAR and temperature rise	Metal object	FEM	8	^(74)^
47	Smondrk *et al.*	CST Microwave Studio	SAR	Metallic Implant	FIT	8	^(75)^
48	Jovanovic *et al.*	CST Microwave Studio	SAR	Effect of metal near body	FIT	7	^(76)^
49	Mydlová *et al.*	CST Microwave Studio	SAR	Railway vehicle	FIT	8	^(77)^
50	Zhou *et al.*	-	SAR, electric and magnetic field	Metro train	FIT	8	^(80)^
51	Wang *et al.*	FEKO & CST Microwave Studio	Magnetic flux and SAR	Subway vehicle	MoM and FIT	8	^(81)^
52	Lieli *et al.*	COMSOL Multiphysics	Electric and magnetic field	Magnetic guided rail	FEM	8	^(83)^
53	Harris *et al.*	Sim4life	Electric field and SAR	Enclosed capsule environment	FDTD	9	^(84)^
54	Colella *et al.* 2021	Sim4Life	Electric field and SAR	Military vehicle	FDTD	9	^(85)^
55	Colella *et al.* 2022	Sim4Life	Electric field	Military vehicle	FDTD	9	^(86)^
56	Benini *et al.*	Sim4Life	SAR	Vehicle	FDTD	8	^(87)^
57	Bacova *et al.*	CST Microwave Studio	SAR	Elevator	FIT	7	^(90)^
58	Karatsi *et al.*	SEMCAD-X	SAR and temperature	Elevator	FDTD	8	^(91)^
59	Celaya-Echarri *et al.* 2019	EME SPY 121 and 3D-Ray Launching algorithm using Matlab	Electric field	Urban transport busses	3D Ray Launching and Exposimeter measurements	9	^(92)^
60	Celaya-Echarri *et al.* 2021	3D Ray Launching simulation, EME SPY EVO and EME SPY 200	Electric field	Shopping malls	3D Ray Launching and Exposimeter measurements	9	^(96)^
61	Celaya-Echarri *et al.* 2022	EME SPY Evolution and 3D ray tracing technique	Power density	Aircraft cabin	3D Ray Launching and Exposimeter measurements	9	^(97)^
62	Göcsei *et al.*	EverTech EMDEX II and COMSOl MultiPhysics	Electric and magnetic field	Industrial power line exposure	FEM and measurement	9	^(98)^
63	Ramos *et al.*	Spectrum analyzer and 3D Ray Launching algorithm	Electric field	Hospital	3D Ray Launching and Spectrum analyzer	9	^(99)^

#### Exposure in indoor environment

Choi *et al.* (1) analyzed RF-EMF exposure data from 91 participants, including parents and children in Seoul, Cheonan, and Ulsan, South Korea, by considering the body shadow effect^(27)^. The participants carried ExpoM-RF for 48 hours, recording values at 4-second intervals. The body shadowing effect was incorporated by a factor of 1.4 to obtain actual exposure values. Results showed that South Korea’s base station RF-EMF exposure levels were higher than those reported in Europe and Australia and also higher inside metro trains than in homes.

Karpowicz *et al.* (2) measured RF-EMF exposure in indoor environments of Warszawa and Madrid, Spain, during crowded and empty periods of shopping malls^(28)^. The RF-EMF exposure in uplink frequency bands of mobile phones was higher in crowded malls than in empty ones. Moreover, the exposure was higher in Madrid than in Warszawa but remained within ICNIRP limits^(7)^.

Zeleke *et al.* (3) recorded WiFi exposure of 63 Australian adults using ExpoM-RF^(29)^. Participants carried PEM in a small hip bag without considering the body shadowing effect. RF-EMF exposure was higher for uplink bands of WiFi than downlink bands and varied throughout the day. However, exposure levels remained significantly below the ICNIRP limits^(7)^. Hardell *et al.* (4) utilized EME SPY 200 to measure RF-EMF exposure in an apartment in Stockholm, Sweden^(30)^. The study found that exposure levels were highest in children’s bedrooms, making them unsuitable for long-term living. However, the study was conducted in 2017 and may not reflect current exposure levels. Additionally, the body shadowing effect was also not considered. Zeleke *et al.* (5) investigated exposure perceptions in relation to RF-EMF exposure from mobile phone base stations^(31)^. The study recruited 383 participants and divided them into three groups: a basic information group, a precautionary group and a personal exposure measurement group. The individuals with access to measurement using PEM had increased confidence in protecting themselves from RF-EMF exposure than other groups.

Massardier-Pilonchery *et al.* (6) investigated RF-EMF exposure among 28 employees in media libraries in Lyon, France^(32)^. The researchers used EME SPY200, spectrum analyzer, isotropic probe and NARDA EHP 200 probe to measure exposure across 87 to 5850 MHz. The results showed that exposure was higher for Walkie-Talkies and depended on the geographic location of the outside source.

Ramirez-Vazquez *et al.* (2019a) (7) conducted a study in Albacete, Spain, to measure RF-EMF exposure across 14 different microenvironments, using four EME SPY 140^(33)^. The results showed that personal exposure was extremely low for all frequencies and was within ICNIRP basic restrictions. However, the primary exposure sources were DECT, followed by mobile phones and WiFi, and reduced the participants’ risk perception about their exposure levels. Similarly, in another study, Ramirez-Vazquez *et al.* (2019b) (8) conducted a study on RF-EMF exposure during temporary events where additional antennas were installed^(34)^. The researchers used two EME SPY 140 mounted on the body and found that the exposure was lower than the ICNIRP guidelines. However, the exposure was higher during the event than before and after.

Iyare *et al.* (2019) (9) investigated the correlation between indoor and outdoor RF-EMF exposure in Leuven, Belgium^(35)^. The results showed that the overall exposure was higher in the outdoor environment compared with the indoor environment due to the shielding effect of walls. Kiouvrekis *et al.* (10) measured RF-EMF exposure in 65 schools in Greece^(36)^. The results indicated no correlation between RF-EMF radiation and distance from the nearest antenna in urban regions. Moreover, the RF-EMF exposure in the range of 27 MHz to 3 GHz was at least 3120 times less than Greek limits and 60% below the ICNIRP limits^(7)^. Loh *et al.* (11) studied RF-EMF exposure in the indoor environment for a massive MIMO system^(37)^. The study involved testing an MIMO system with 128 channels at 2.63 GHz, with up to 864 allocated resource blocks for four simultaneous active beams. The results indicated that increasing the beams and allocated resources resulted in higher RF-EMF exposure.

Paniagua *et al.* (12) concluded that FM broadcasting was the major contributor to RF-EMF exposure in indoor and outdoor environments. However, the contribution from mobile communication antennas was higher in the outdoor environment^(38)^. Keshmiri *et al.* (13) found that the measured PD of the outdoor station was less than 1% of the limit and higher on the roof and loft^(39)^. Moraitis *et al.* (14) studied RF-EMF exposure in different corporate building floors and found that the exposure values were higher for mobile communication, increased linearly with floor levels and were below the legislated exposure limits^(40)^. Mannan *et al.* (15) measured RF-EMF exposure in 16 different buildings and found that PD values were higher in hospitals and lower in offices^(41)^. Ramirez-Vazquez *et al.* (2020) (16) analyzed RF-EMF exposure for indoor and outdoor buildings at a Spanish school and recorded higher exposure levels for WiFi outside the school building on weekends, whereas higher inside the school building during weeks^(42)^. Onishi *et al.* (17) evaluated RF-EMF exposure in outdoor and underground shopping malls in Japan and found that the exposure level was higher in urban than suburban environments and differed by 7 dB^(43)^. Iyare *et al.* (18) compared indoor mobile phone exposure for data transmission and voice calls, respectively, and found that the electric field strength for 4G was higher compared with 2G and 3G^(44)^. Ramirez-Vazquez *et al.* (19) assessed the exposure level of 2G and 5G WiFi frequency bands and found that the WiFi-2G exposure was higher but was below ICNIRP basic restrictions^(45)^. Similarly, Kapetanakis *et al.* (20) found that the main contribution to the exposure was from mobile communication and broadcasting frequencies, and it was higher in urban than suburban environments due to the higher number of devices^(46)^. Rathebe *et al.* (21) determined RF-EMF in two unoccupied residential apartments near the base station using a TM196 RF field strength meter^(47)^. The peak exposure was observed in the lounge area of residential building two and lowest in the balcony of building one.

Bakcan *et al.* (22) conducted a study to measure RF-EMF exposure at the Bursa Uludag University Görükle campus in Turkey using SRM-3006 and machine learning techniques, respectively^(48)^. Their results indicated that the ANN-1 method could predict RF-EMF exposure 7.5% better than the ANN-2 method. However, the exposure levels were lower than the regulatory threshold, with an average exposure of 1.024 V m$^{-1}$. Schmutz *et al.* (23) investigated RF-EMF exposure in 148 adolescents living in and around Greater London using ExpoM-RF^(49)^. The results showed that adolescents in London had higher exposure levels than those in other European countries, with downlink sources having higher exposure levels. Similarly, Hamiti *et al.* (24) studied RF-EMF exposure inside and outside a primary school in Kosovo using the EME SPY 140^(50)^. The results indicated that mobile phones were the primary contributor to exposure, with higher average PD inside the school building during weekdays. Additionally, outdoor exposure levels were higher than inside exposure levels.

Ramirez-Vazquez *et al.* (2023) (25) compare RF-EMF exposure levels in different frequency bands in 2022 with those in 2017, 2018 and 2019 in various settings^(51)^. The results showed that exposure levels were higher in classrooms with students and lowest in the professor’s room. However, the authors did not thoroughly compare the results for all past years. Furthermore, the comparison showed a decrease in exposure values from 2017 to 2019, which contradicts the claim that exposure levels would increase with the increase in the number of devices.

#### Exposure in occupational environment

Measuring RF-EMF exposure in different occupational work sites is crucial in identifying potential long-term effects where workers spend up to 8 hours daily. Michalowska *et al.* (26) used the ESM-140 PEM to measure exposure in the working area of CNC machines for mobile phone frequencies^(52)^. The results suggested that electric field strength was lower for all inferred frequencies and locations. However, it is important to note that the study did not account for the body shadowing effect, and there was a lack of statistical analysis performed on the collected data.

Similarly, Cerezci *et al.* (27) evaluated RF-EMF exposure in 10 different indoor environments of factory workers for low- and high-frequency sources^(53)^. The exposure was higher for two spots according to BTK (National Information Technology and Communication Authority (Turkey) limits). It was recommended to take appropriate precautions to reduce the exposure level of workers. Wafik *et al.* (28) investigated RF-EMF exposure in different office spaces in New York City^(54)^. The exposure levels varied depending on the distance from the source, with the hotspot near the router having the highest exposure value. It is crucial to take appropriate precautions, such as maintaining a safe distance or using protective measures, to minimize potential exposure levels.

#### Exposure in transport and metallic environments

Proximity to metallic structures near the source of RF-EMF can increase potential exposure in confined microenvironments, such as public transport and subway trains. Sagar *et al.* (29) compared RF-EMF exposure in 94 different microenvironments and 18 public transport vehicles across six countries^(55)^. Mobile phones and broadcasting primarily contributed to the exposure, tended to increase in urban areas, and was highest in buses and trains in Switzerland.

Gryz *et al.* (30) investigated RF-EMF exposure of WiFi in trains^(56)^. The measurements showed that the exposure in the train was higher than in a laboratory due to reflections and higher in crowded trains than in trains with fewer passengers. Similarly, Gombarska *et al.* (31) assessed exposure to mobile phones and Bluetooth inside personal vehicles in an urban environment^(57)^. The study showed that the electric field strength was higher by 10 times in the vehicle’s cabin. Sen *et al.* (32) experimentally analysed exposure in the anechoic chamber at 835 MHz for goat tissues^(58)^.

Due to the lack of standardized procedures for broadband measurements of RF-EMF exposure, different methods have been proposed, providing new research opportunities^(59)^. Schilling *et al.* (33) measured RF-EMF exposure in connected vehicles for nine different positions^(60)^. The ITS-G5 variant of the antenna generated the highest electric field strength and required continuous monitoring as the number of users increased. The highest field strengths occurred near the transmitting antenna. Vaverka *et al.* (34) investigated RF-EMF exposure in elevators and inside cars using a dipole antenna and spectrum analyzer^(61)^. The measurements showed that exposure was higher inside the elevator than outside and higher during communication. Therefore, avoiding using mobile phones in shielded and enclosed environments is recommended.

#### Exposure measurement using body worn distributed exposure meters

Several body-worn distributed exposure meters (BWDMs) have been developed to evaluate personal RF-EMF exposure in indoor environments. In particular, Aminzadeh *et al.* (35) introduced a new BWDM for personal exposure assessment^(62)^. The BWDM presented by Aminzadeh *et al.* comprises 22 nodes distributed over the torso and embedded on a jacket to measure incident PD in the range of 790–5513 MHz. The maximum difference anticipated by the BWDM was 3.2 dB and validated for five different frequency bands and indoor environments. Huss *et al.* (36) compared the RF-EMF exposure measured by the BWDM with ExpoM-RF and EME SPY200^(63)^. The same BWDM used by Aminzadeh *et al.* was employed to measure exposure in Belgium, Spain, France, the Netherlands and Switzerland. The downlink band of all frequencies was found to be the major contributor to RF-EMF exposure. However, the BWDM required calibration before each measurement campaign, necessitating more preparation time. In addition, the BWDMs were limited by their heavy weight (about 4 kg) and bulkiness, which can be challenging for long-term monitoring.

#### Exposure assessment using interpolation

Bustillo *et al.* (37) utilized the Shepard interpolation method to estimate RF-EMF exposure indoors using NARDA SRM-3000^(64)^. However, the interpolation methods produced varying errors due to reflections, diffraction and scattering of the radiations. Martínez-Gonzalez *et al.* (38) used the Kriging interpolation procedure to create an electric field indoor map of the library reading room of the Telecommunication Engineering School, ETSIT, at Universidad Polit’ecnica de Cartagena by measuring exposure at 386 different locations^(65)^. The study implemented the Elimination of Less Significant Points and Geometrical Elimination of Neighbors strategies to reduce the number of measuring points and increase the accuracy of the exposure map. In contrast, Kljajic *et al.* (39) proposed an adaptive boundary approach for broadband and continuous monitoring of RF-EMF exposure that utilizes the daily lower and upper exposure boundaries and their adaptation based on the spectral characteristics of the measurement location^(66)^. Measurements were conducted using NARDA NBM-550 at the University of Novi Sad campus, adopting global exposure ratio^(66, 67)^. The study showed that the adaptive approach resulted in more appropriate reference levels, reducing the difference by 36.25%.

### Dosimetry numerical evaluation

A total of 23 articles (40 to 63) evaluated RF-EMF exposure in indoor and confined environments using numerical dosimetry. The indoor/dwelling includes RF-EMF exposure analysis in all indoor environments, while the confined environment includes evaluations in trains, buses, military vehicles, metallic objects near RF-EMF sources and human bodies as summarized in Table 2. Additionally, five selected articles (listed in Table 2 as numbers 59–63) used both dosimetric measurement and numerical simulation to assess RF-EMF exposure in different confined environments.

#### Exposure in indoor and confined environments

Bejenaru *et al.* (2019) (40) evaluated the SAR in the human body inside a room under different scenarios^(68)^. The room was designed with dimensions of 800 $\times $ 800 $\times $ 30 cm, and various building materials were considered. The SAR was significantly lower than the ICNIRP, IEEE and EU standards. However, a homogeneous model limits the exposure investigation in different tissues. Similarly, Psenakova *et al.* (41) investigated SAR using a dipole antenna on the head in both shielded and open space scenarios^(69)^. The SAR was higher in the shielded environment than in open space due to reflections.

Ashraf *et al.* (42) investigated SAR for wearable dipole antennas in different underground mine scenarios at 868 and 2400 MHz^(70)^. The study investigated different underground mine tunnel scenarios using a concrete structure and metal steel arch. The wearable dipole antenna was mounted on the head, outside the safety helmet and on the torso. The SAR was negligibly higher in underground mine scenarios than in open space. It was maximum for the arch scenario and was below the basic restriction of ICNIRP. It was suggested that placing an antenna outside the safety helmet shields the head from RF-EMF exposure. However, this study did not implement a realistic wearable device or consider the rocks’ properties based on different geology. It emphasized the need for further research on RF-EMF exposure in subterranean environments considering real environmental parameters.

Chiaramello *et al.* (43) assessed WiFi exposure in a realistic apartment using principal component analysis and Gaussian process regression^(71)^. The results showed that the exposure was lower than the ICNIRP basic restrictions, but no comparison was made for the indoor and free-space environments. Shikhantsov *et al.* (44) presented a numerical approach to estimate RF-EMF exposure in indoor industrial environments using ray tracing and modified finite domain time domain techniques^(72)^. The maximum transmitted power was found to be 35 and 110 W for single transmission in line of sight and non-line of sight propagation, respectively. Bejenaru *et al.* (45) evaluated SAR for a custom model in a room made of loamy and concrete material^(73)^. The study considered plane wave exposure without comparing free space and indoor environment exposure.

#### Exposure assessment in close proximity of metallic objects

Numerous studies have investigated how metallic objects affect human exposure to RF-EMF^(74, 75, 76)^. Bhargava *et al.* (46) investigated the SAR and temperature elevation in a heterogeneous model in the presence of different metallic objects close to the 1800 MHz^(74)^. The results suggested that all shapes of metals (metal pin, spectacle and ring) increase the SAR and temperature in the head tissues. Similarly, Smondrk *et al.* (47) investigated the impact of metallic pacemaker implants as a function of distance and frequency^(75)^. The SAR was higher in the presence of a needle implant, and absorption was higher in the superficial tissues of the human body. The safety distance for 900 MHz was 55 mm for needle implants. Jovanović *et al.* (48) investigated the electric field and SAR using a PIFA antenna in the case of metal (aluminum) frame glasses^(76)^. The glasses lowered the electric field by four times, indicating that the glasses shield the head from RF-EMF exposure.

#### Exposure in transport and metallic surroundings

Mydlová *et al.* (49) numerically analyzed the exposure of mobile phone frequencies in a railway vehicle on a human body with a cochlear implant^(77)^. The study includes PIFA at 900 and 1800 MHz with an output power of 0.5 and 1 W, respectively. A cochlear implant consisting of an external microphone, a speech processor and a connecting cable is used^(78)^. The cochlear implant was modeled using various materials^(79)^, and the railway vehicle was modeled with a metal frame. The mobile phone was mounted on the human model at a distance of 17 and 22 mm. The results indicated that metallic implants alter the electric field strength in the tissues, and SAR was 10% higher than in open space. The recommended safe distance from the body should not be less than 10 mm.

Zhou *et al.* (50) evaluated the shielding effectiveness of metro cabins to protect drivers from RF-EMF exposure^(80)^. The SAR was analyzed inside and outside a metro cabin, modeled with aluminum alloy and glass windows. External EMF was introduced via a Terrestrial Trunked Radio (TETRA) system antenna (835 MHz), while an internal EMF source was the Communication-Based Train Control (CBTC) system antenna. Without AI alloy, the TETRA system’s electric field peak in the cab was 322 times higher than with AI alloy, resulting in effective shielding. Conversely, for the CBTC system, the electric field in the AI alloy cabin was twice as high as without alloy, indicating increased driver exposure.

Wang *et al.* (51) investigated the influence of different materials and shielding on RF-EMF exposure inside subway trains^(81)^. The results showed that the magnetic flux in the train depended on the material type used, windows and shielding layers. The magnetic flux for aluminum alloy, carbon fiber and stainless steel was found to be 6.3, 5.8 and 5.4% of ICNIRP limits, respectively. In a related study, Wang *et al.* recommended using various antennas and external intentional electromagnetic pulses on different warships, vehicles and airplanes to reduce the risk of long-term exposure^(82)^. Similarly, Lieli *et al.* (52) also studied the exposure from permanent magnet-guided rails for different human body positions^(83)^. The results showed that the magnetic flux in the body decreased as a function of the distance between the body and the source.

Harris *et al.* (53) investigated SAR for various positions in an enclosed cylindrical capsule environment^(84)^. Results showed that the signal distribution varies with the distance from the transmitter. However, the exposure can be higher in a real capsule environment due to the presence of more than one person.

Colella *et al.* (2021) (54) simulated low-frequency exposure for different scenarios, including a vehicle with an open manhole, a vehicle with the operator partially outside the manhole, wearing a protective helmet and the cabled headset^(85)^. The results revealed that the electric field in the head of the operator with the helmet was 10% higher when the operator was partially outside the manhole. However, the local SAR values inside the human body were a maximum of 14 mW kg$^{-1}$, and the whole-body SAR value averaged over 10 g of tissue was below corresponding limits. In another study, Colella *et al.* (2022) (55) evaluated RF-EMF exposure in the military vehicle for different personal protective equipment and posture^(86)^. The results showed that placing the operator’s arm on the vehicle increased the SAR from 0.43 to 2.03 mW kg$^{-1}$. However, the human body exposure in this study was in line with guideline limits of military applications^(17)^.

Benini *et al.* (56) utilized numerical dosemeters to investigate the exposure of pedestrians to vehicle-to-vehicle (V2V) communication^(87)^. Two V2V monopole antennas were used with an input power of 1 W mounted on the roof, windscreen and back of the vehicle. The human body was positioned in five locations: front, back, front-side, middle-side and back-side of the car using the Huygens box approach^(88, 89)^. The results showed that the SAR value was less than ICNIRP thresholds for all cases. However, the front orientation of the antenna consistently yielded a higher level of exposure.

Bacova *et al.* (57) studied the SAR in the chest of children and adults in different scenarios, including open space, cellar and inside the subway cabin^(90)^. The SAR was higher in the chest of toddlers than in adults in all scenarios, which could pose a risk to children’s health development. Similarly, Karatsi *et al.* (58) investigated the SAR and temperature rise in the tissues of pregnant women and a child using a metallic elevator at 1000 and 1800 MHz^(91)^. Their simulation results showed that a child’s exposure was significantly affected by the position of the phone and less affected by the position of the near human body. However, the opposite was observed in the case of pregnant women.

Various studies have employed dosemeter numerical and measurement methodologies to evaluate RF-EMF exposure in confined environments. In particular, Celaya-Echarri *et al.* (2019) (59) compared electric field distribution for measured and simulated scenarios in different types of urban transportation^(92)^. The EME Spy 121 was placed closest to the user in static and dynamic positions; the highest level of RF-EMF exposure was observed during a phone call^(93)^. A 3D-Ray tracing algorithm was utilized to investigate RF-EMF exposure inside two types of urban buses, the articulated and rigid bus^(94)^ and cars^(95)^. Results showed that electric field values were higher in the rigid bus and had a significant influence due to the shielding effect of the body.

Celaya-Echarri *et al.* (2021) (60) examined RF-EMF exposure in five different shopping malls located in different countries using the PEM and the 3D-ray tracing method^(96)^, previously explained in reference^(92)^. They found that the highest peak exposure was observed in indoor shops in Madrid, followed by general corridors in Warszawa. Similarly, Celaya-Echarri *et al.* (2022) (61) conducted another study to evaluate RF-EMF exposure for passengers inside aircraft cabin environments using the same 3D-ray tracing technique^(97)^. The results indicated that narrower aircraft showed higher exposure levels than broader ones.

Gocsei *et al.* (62) also investigated extremely low-frequency exposure risks in the powerhouse of an industrial environment in Hungary^(98)^. The results indicated that the electric and magnetic field strengths remained below current limits. Furthermore, Ramos *et al.* (63) conducted measurements and simulations of RF-EMF exposure of the Radio-Frequency IDentification readers in the hospital and an echoic chamber^(99)^. It is important to consider preventive measures to reduce thermal effects.

## Discussion

The dosimetric data comparison between confined and open spaces is detailed in Table 3. The results reveal elevated exposure metrics in open spaces attributed to the outdoor (open space) RF-EMF sources. Conversely, in scenarios involving mobile RF-EMF sources, exposure metrics are higher in confined environments than in open spaces.

**Table 3:** List of studies compare dosimetric data between confined and open spaces

**Study/ Source**	**Type of environment**	**Frequency**	**Dosimetric data**	
			**Confined**	**Open space**
Choi *et al.*^(27)^	Metro & outside	87.5–5875 MHz	4725.9 $\mu $W m$^{-2}$	496.5 $\mu $W m$^{-2}$
Hardell *et al.*^(30)^	Bedroom & balcony	900 & 1800 MHz	10.7 $\mu $W m$^{-2}$	82.4 $\mu $W m$^{-2}$
Ramirez Vazquez *et al.* 2019a^(33)^	Home & outdoors	1800 MHz	247.1 $\mu $W m$^{-2}$	143.2 $\mu $W m$^{-2}$
Iyare *et al.* 2019^(35)^	indoor & outdoor	900 MHz	0.83 V m$^{-1}$	1.80 V m$^{-1}$
Paniagua *et al.*^(38)^	indoor & outdoor	900 MHz	0.027 V m$^{-1}$	0.176 V m$^{-1}$
Ramirez Vazquez *et al.* 2020^(42)^	indoor & outdoor	2400 MHz	25.3 $\mu $W m$^{-2}$	13 $\mu $W m$^{-2}$
Onishi *et al.*^(43)^	Underground malls and	800 MHz	7 dB	7.5 dB
	outdoor	2100 MHz	8.1 dB	8.3 dB
Ramirez Vazquez	Workspace & outdoor	2400 MHz	499.7 $\mu $W m$^{-2}$	239.4 $\mu $W m$^{-2}$
2021 *et al.*^(45)^		5150–5850 MHz	264.9 $\mu $W m$^{-2}$	178.7 $\mu $W m$^{-2}$
Hamiti *et al.*^(50)^	indoor & outdoor	900 MHz	1932 $\mu $W m$^{-2}$	576 $\mu $W m$^{-2}$
Sen *et al.*^(58)^	metallic chamber & free space	850 MHz	3.5 V m$^{-1}$	$\approx $ 3.8 V m$^{-1}$
Vaverka *et al.*^(61)^	Elevator & open space	1800 MHz	12.2 $m$V m$^{-1}$	6.8 $m$V m$^{-1}$
Psenakova *et al.*^(69)^	shielded room & open space	900 MHz	18.05 W kg$^{-1}$	16.01 W kg$^{-1}$
Ashraf *et al.*^(70)^	Underground mine & open	2400 MHz	1.6 W kg$^{-1}$	1.5 W kg$^{-1}$
	space	868 MHz	0.47 W kg$^{-1}$	0.42 W kg$^{-1}$
Bacova *et al.*^(90)^	Railway wagon & open space	900/1800 MHz	1.61 W kg$^{-1}$	0.9 W kg$^{-1}$

**Figure 2:**
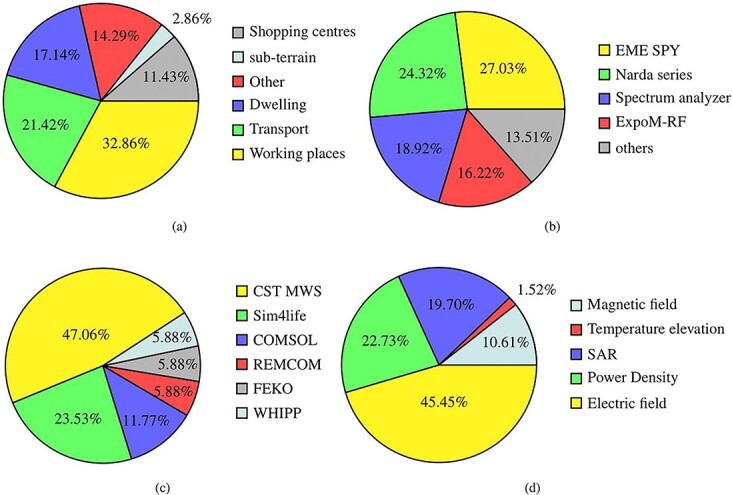
Pie chart of studies considered different (a) indoor environments, (b) exposimeters, (c) simulation software and (d) exposure parameters.

Various confined environments were assessed, as depicted in Figure 2a. Most studies focused on evaluating RF-EMF exposure in dwellings and working spaces; however, few studies evaluated RF-EMF exposure in subterranean environments.

Various exposimeters were utilized to measure RF-EMF exposure in different microenvironments, as shown in Figure 2b. The ExpoM-RF, EME SPY and Narda series were the most commonly used exposimeters. These exposimeters were carried by researchers or participants to measure real RF-EMF exposure in the environment, but the body shielding effect is a significant source of uncertainty^(100)^. In numerical dosimetric studies, the most commonly used software was CST MWS^(101)^, as shown in Figure 2c, followed by Sim4life^(102)^ and COMSOL^(103)^. The electric field was the most commonly used parameter for investigating RF-EMF exposure using dosimetry simulation, as shown in Figure 2d. The temperature elevation in tissues is another crucial parameter to be considered for evaluating RF-EMF exposure, incorporating the thermal properties of tissues.

RF-EMF exposure in sub-terrain environments such as underground mines, subway stations and underground malls has received less attention in the literature. Underground mines pose the greatest challenge among these environments due to their confined and harsh nature and workers’ long-term use of wearable devices. Wearable devices have been extensively researched for their potential application in underground mines^(104, 105)^. However, chronic implementation of wearable devices in such harsh environments can lead to health concerns. Recently, Ashraf *et al.* conducted a pilot study that did not consider the underground mine environment’s high temperature^(70)^. Therefore, assessing long-term workers’ exposure to RF-EMF in high-temperature environments is essential for different occupational settings.

## Challenges and emerging directions

The lack of standard procedures to measure personal RF-EMF exposure leads to inconsistent and inaccurate assessments of potential vulnerability. Thus, there is a critical need to establish uniform standards for a personal assessment of RF-EMF exposure, considering the body shadowing effect. There is also a need for more research to understand the long-term effects of RF-EMF exposure, particularly in high-risk populations such as children and pregnant women.

The rapid proliferation of wireless devices, particularly 5G networks, poses a significant challenge for monitoring and managing RF-EMF exposure levels in general and occupational settings. With an increase in the number of 5G devices, the potential exposure of humans in confined and reflective environments also increases, necessitating investigation of RF-EMF exposure in such environments. However, different standard procedures were also developed by regulatory bodies^(106, 107)^. The updated exposure limits, metrics and procedures are primarily for laboratory settings, with no established procedures for personal exposure assessment.

V2V communication is exchanging information between two or more vehicles using wireless communication. Currently, there is limited research on the effects of V2V communication on RF-EMF exposure, and more studies are needed to understand the potential risks and develop effective mitigation strategies.

Few studies investigated temperature elevation in tissues in indoor and confined environments. There is a need to study the impact of temperature elevation on tissues in different confined environments, considering various temperatures, duration and surrounding environments.

Machine learning (ML) algorithms have been implemented for predicting RF-EMF exposure and can predict the relationship between various RF-EMF exposure metrics and reduce the computation time required. Therefore, further research should focus on evaluating the effectiveness of different ML techniques in measuring and predicting RF-EMF exposure, which can ultimately enhance the accuracy and efficiency of RF-EMF exposure assessment.

## Conclusion

In this study, we conducted an SLR of 63 articles related to RF-EMF exposure in confined environments. The articles were selected based on a QA and divided into measurement and simulation studies. Our literature analysis demonstrates that RF-EMF exposure is higher in working spaces and transport than in dwellings, largely due to multiple exposure frequencies and excessive users. However, evaluating sub-terrain environments remains limited despite the growing use of wearable wireless devices in these settings. We also identified the different exposimeters and numerical dosimetry software tools commonly used for measuring and simulating RF-EMF exposure in confined environments. Furthermore, we found that the electric field is the most commonly investigated parameter for evaluating RF-EMF exposure using dosimetry simulation and measurement in confined environments. Given the increasing use of wearable wireless devices in sub-terrain and other occupational environments, there is a critical need to evaluate RF-EMF exposure in high-temperature environments, particularly with respect to temperature elevation in tissue. Future studies should evaluate the potential health impacts of long-term exposure to RF-EMF, especially in occupational settings where workers are exposed for extended periods.
